# Methylation data of mouse Rb-deficient pineoblastoma

**DOI:** 10.1016/j.dib.2020.106229

**Published:** 2020-08-25

**Authors:** Philip E.D. Chung, Eldad Zacksenhaus

**Affiliations:** a Toronto General Research Institute - University Health Network, 67 College Street, Toronto, ON M5G 2M1, Canada; b Laboratory Medicine & Pathobiology, University of Toronto, Toronto, ON, Canada

**Keywords:** Pineoblastoma, Methylation sequencing, Mouse models, Rb, p53

## Abstract

Methylation profiling is widely used to study tumor biology and perform cluster analysis, particularly in brain cancer research where tissue biopsies are scarce. We have recently reported on the development of novel mouse models for germ line mutations in pineoblastoma (*Nature Communications*, 2020). Here, we present unpublished methylation profiling of 8 Rb-deleted/p53-deleted pineoblastoma from our mouse model as well as 3 normal cerebellum tissues as control. The primary dataset can be accessed via SRA (PRJNA638504). These methylation data can be used to perform inter- and intra-species comparisons with other brain cancers as well as with specific subtypes of pineoblastoma, and to investigate potential epigenetic mechanisms and pathways underlying Rb-deficient pineoblastoma-genesis..

**Specifications Table**

**Table:** 

Subject	Cancer Research
Specific subject area	Pediatric brain tumor, mouse pineoblastoma
Type of data	Methylation sequencing read (raw) and Figures
How data were acquired	Reduced Representation Bisulfite Sequencing using Illumina HiSeq 2500
Data format	Raw
Parameters for data collection	Methylation sequencing was performed on genomic DNA collected from mouse Rb/p53-deficient pineoblastoma and normal age-matched mouse cerebellum
Description of data collection	Rb-deficient pineoblastoma were collected from WAP-Cre:Rb^f/f^:p53^f/f^ mice and normal cerebellum samples.. Genomic DNA was extracted using DNeasy Blood & Tissue Kit and was subjected to Methyl-MiniSeq methylation sequencing (Zymo Research, California, USA)
Data source location	University Health Network Toronto, Ontario Canada
Data accessibility	Repository name: Sequence Read Archive Data identification number: PRJNA638504 Direct URL to data: https://www.ncbi.nlm.nih.gov/bioproject/PRJNA638504
Related research article	Philip E. D. Chung, Deena M. A. Gendoo, Ronak Ghanbari-Azarnier, Jeff C. Liu, Zhe Jiang, Jennifer Tsui, Dong-Yu Wang, Xiao, Bryan Li, Adrian Dubuc, David Shih, Marc Remke, Ben Ho, Livia Garzia, Yaacov Ben-David, Seok-Gu Kang, Sidney Croul, Benjamin Haibe-Kains, Annie Huang, Michael D. Taylor & Eldad Zacksenhaus. Modeling germline mutations in pineoblastoma uncovers lysosome disruption-based therapy. *Nature Communications*. 11, 1825 (2020).https://doi.org/10.1038/s41467–020–15585–2

## Value of the Data

• Pineoblastoma is a rare but lethal disease. We have generated mouse models for germ line mutations that drive pineoblastoma and performed RNA-based cluster analysis. Here we provide unpublished DNA methylation data from a mouse model of RB-deficient pineoblastoma.
• This methylation dataset can benefit investigators of brain cancer, pineoblastoma, Rb and p53 tumor suppressors, and metastatic dissemination.
• The methylation dataset can be further used for clustering analysis, discovering *de novo* epigenetic changes, investigating known methylation sites, pathway analysis and therapeutic development.

## 1 Data Description

### 1.1 Methylation profiling of a mouse model of RB-deficient pineoblastoma

Pineoblastoma (PB) is a pediatric brain cancer of the pineal gland with several distinct subtypes, including germline mutations in the tumor suppressors RB or Dicer [1]. We have recently established mouse models for germline PB by disrupting Rb (or Dicer1) together with p53 via the WAP-Cre delete line [2]. Through transcriptomic profiling of brain tumors, we clustered the Rb/p53-deficient mouse model of PB close to human PB as well as to Group 3 and Group 4 MBs, but relatively far from GBM [2], as is the case in human brain tumors [3,4]. We have also generated methylation profiling by Methyl-MiniSeq (Zymo Research) sequencing of Rb/p53-deficient mouse pineoblastoma and normal cerebellum. The raw dataset was deposited in the NCBI Sequence Read Archive under PRJNA638504.

**Fig. 1:**
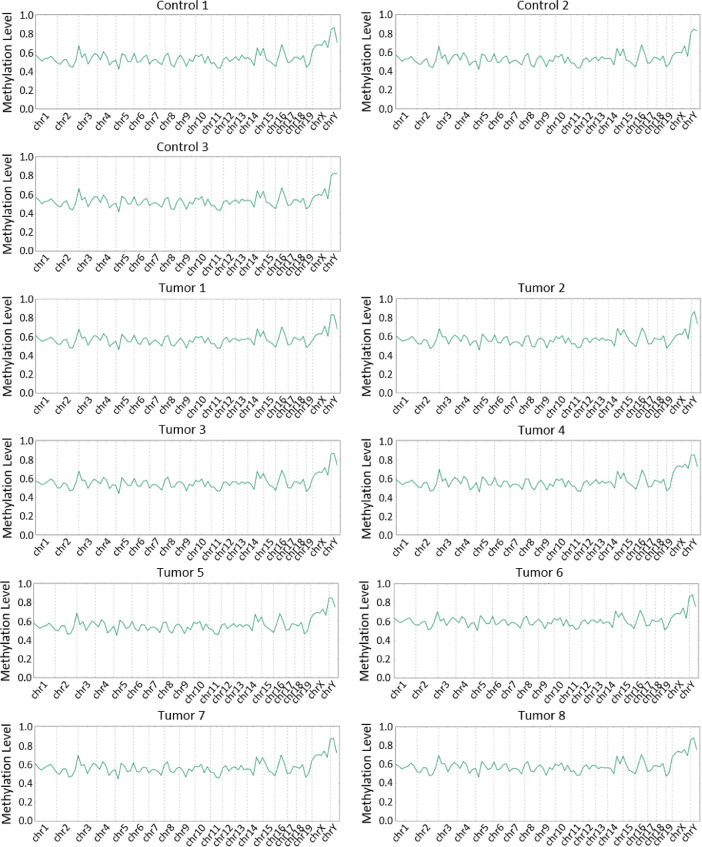
Overall methylation level profiles per chromosome in 8 Rb-deficient pineoblastomas and 3 cerebellum controls.

Fig. 1 shows overall methylation levels of 8 Rb-deleted/p53-deleted pineoblastoma and 3 controls.

**Fig. 2:**
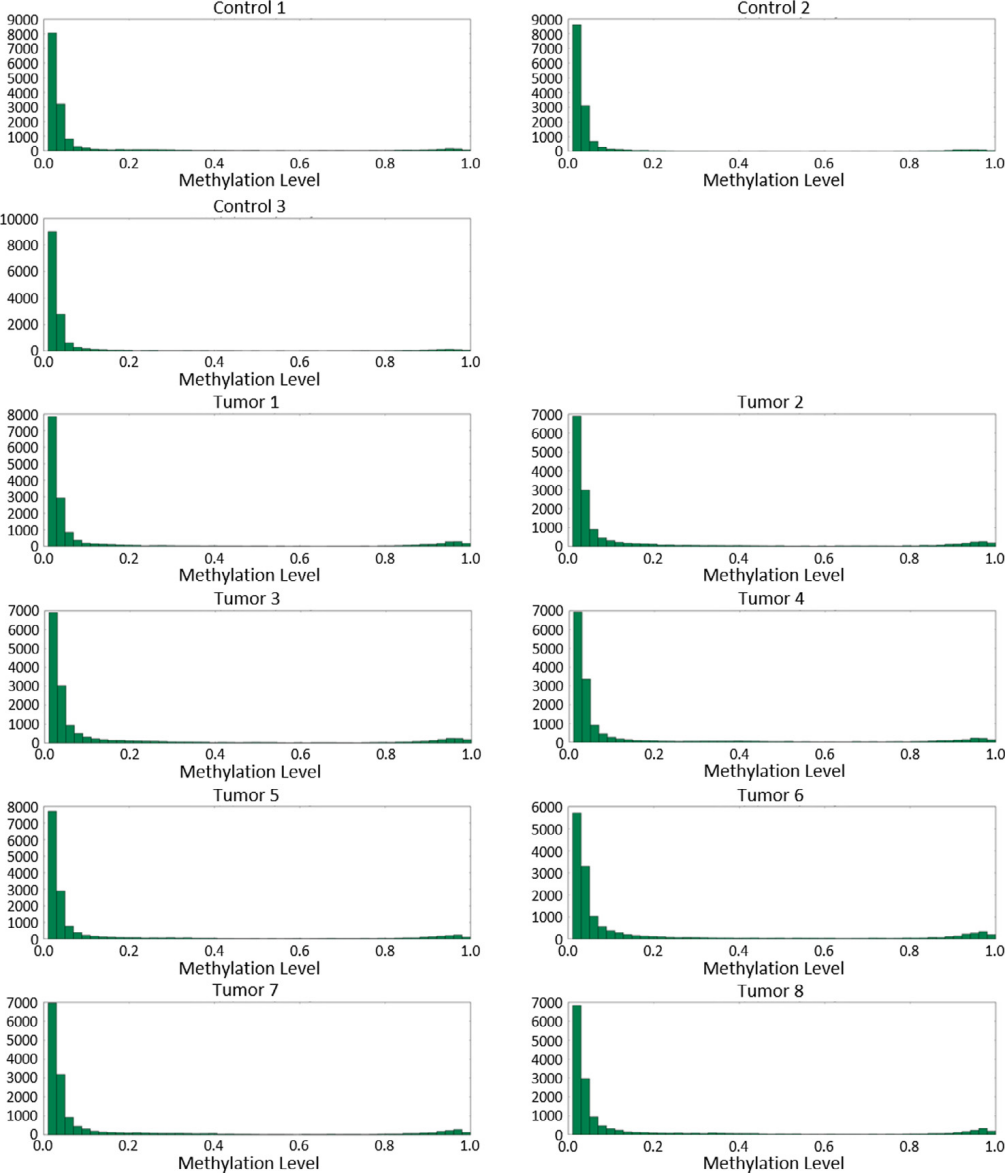
A histogram summarizing the number of sites of CpG at each methylation ratio level in the context of CpG islands.

**Fig. 3:**
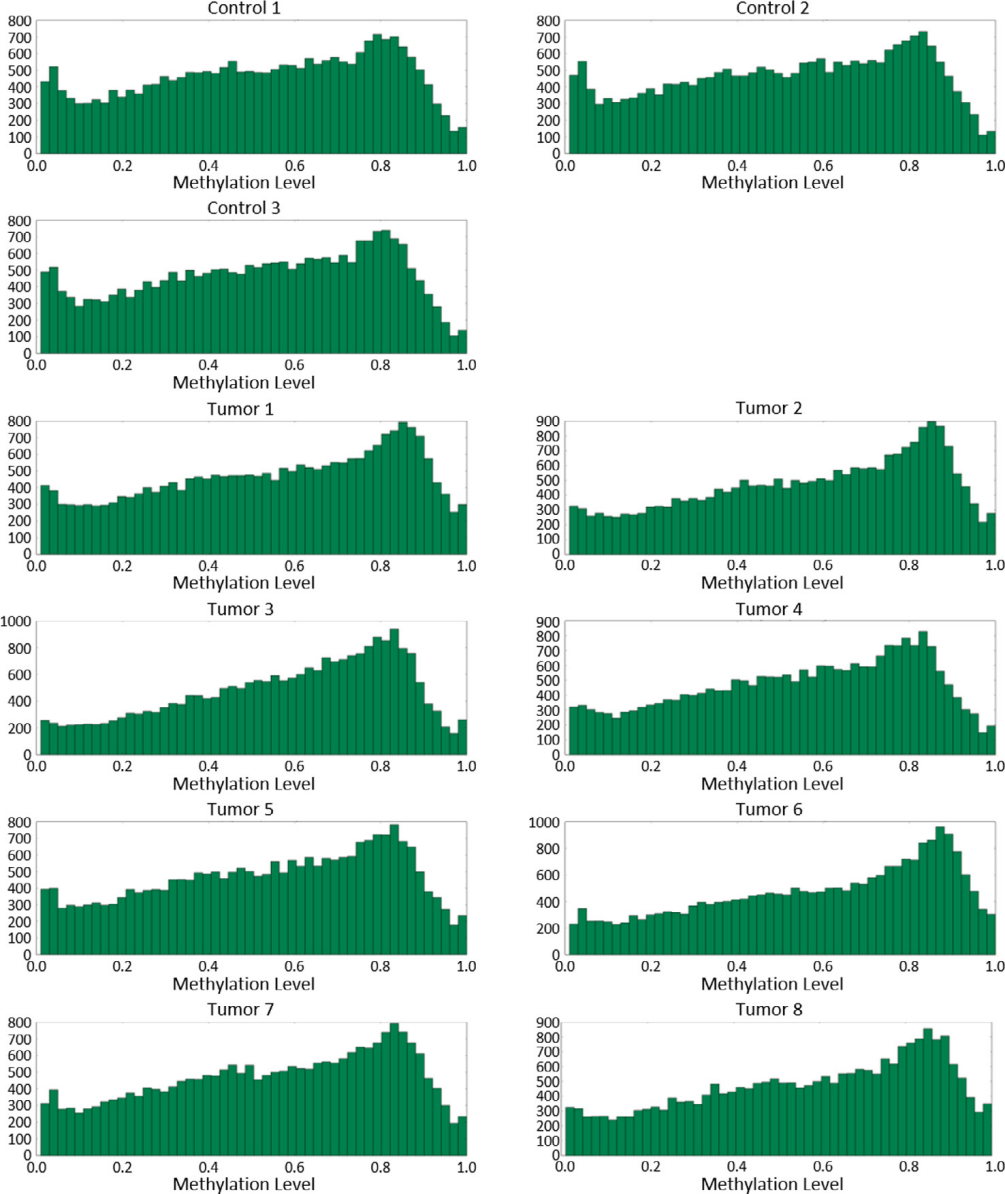
A histogram summarizing the number of sites of CpG at each methylation ratio level in the context of gene bodies.

**Fig. 4:**
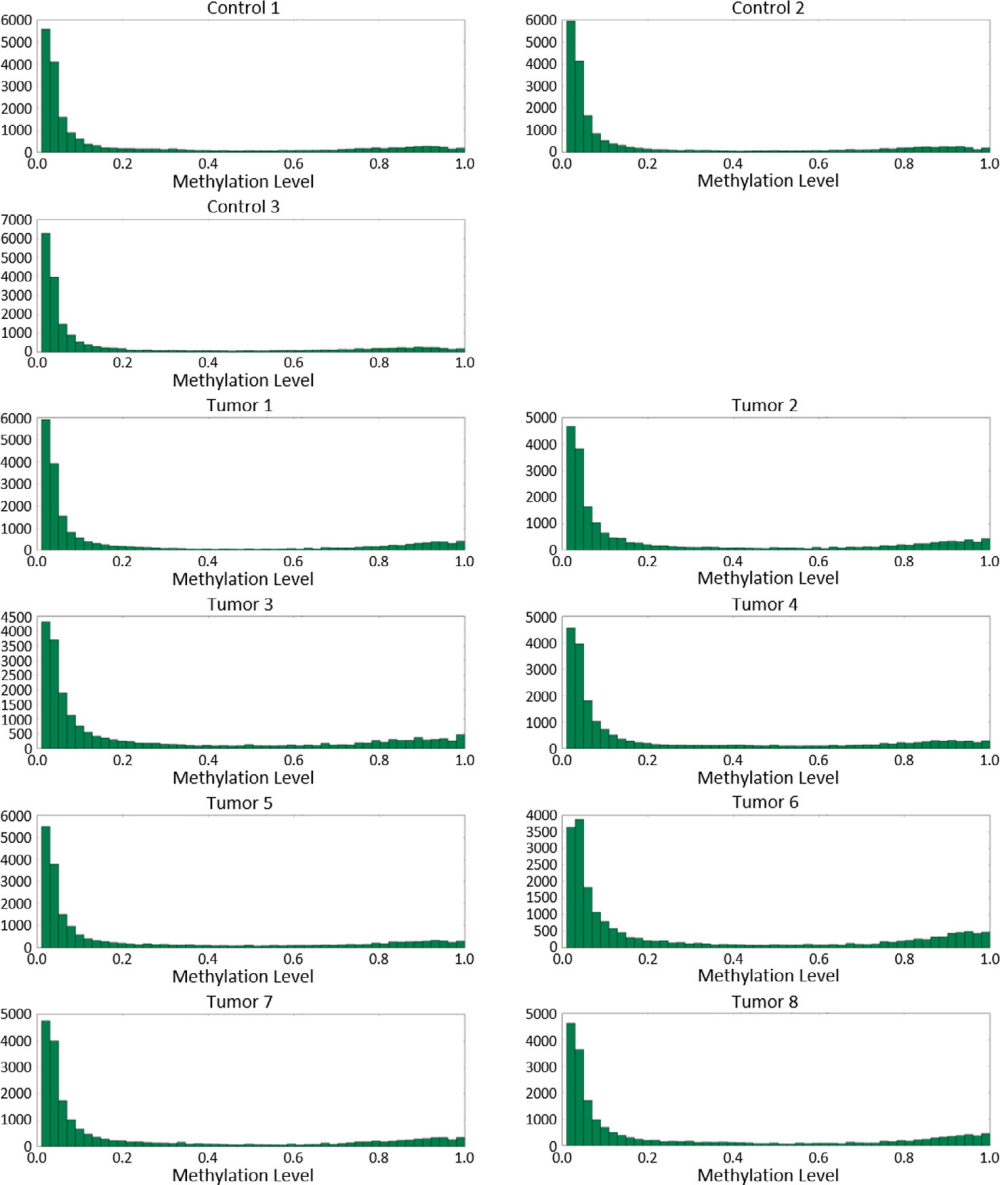
A histogram summarizing the number of sites of CpG at each methylation ratio level in the context of gene promoter.

Figs. 2, Fig. 3, Fig. 4 show CpG island methylation levels, CpG gene methylation levels, and CpG promoter methylation levels, respectively.

## 2 Experimental design, materials and methods

### 2.1 Methyl-MiniSeq methylation sequencing

Tumor and tissue samples were collected from 8 Rb-deleted/p53-deleted pineoblastoma and 3 normal cerebellum as control. Genomic DNA was extracted using DNeasy Blood & Tissue Kit (Qiagen) according to manufacturer's instruction. DNA samples (50 – 200 ng/µl in 40 µl) were subjected to Methyl-MiniSeq methylation sequencing (Zymo Research (California, USA)). 200 – 500 ng of genomic DNA was used for library construction and sequencing. Genomic DNA was fragmented using TaqαI and MspI, ligated to adapters containing 5′-methyl-cytosine, bisulfite-treated using EZ DNA Methylation-Lightning Kit (Zymo Research), and PCR amplified for sequencing on Illumina HiSeq 2500. Sequence reads were identified using Illumina base-calling software and aligned using Zymo Research proprietary analysis pipeline.

## Ethics statement

All mouse experiments were performed in accordance with the current Canadian Animal Care Council guide for the care and use of laboratory animals and were approved by the Toronto General Research Institute Animal Research Committee, UHN.

## Declaration of Competing Interest

The authors declare that they have no known competing financial interests or personal relationships, which have, or could be perceived to have influenced the work reported in this article.
